# Co-design of a single session intervention chatbot for people on waitlists for eating disorder treatment: a qualitative interview and workshop study

**DOI:** 10.1186/s40337-025-01225-x

**Published:** 2025-03-11

**Authors:** Gemma Sharp, Bronwyn Dwyer, Jue Xie, Roisin McNaney, Pranita Shrestha, Christopher Prawira, Anne Nileshni Fernando, Kathleen de Boer, Hao Hu

**Affiliations:** 1 Department of Neuroscience, Monash University, 99 Commercial Road, Melbourne, VIC 3004 Australia; 2 Department of Human Centred Computing, Monash University, Melbourne, Australia

**Keywords:** Eating disorder, Mental health, Single session intervention, Chatbot, Conversational agent, Artificial intelligence, Digital health

## Abstract

**Background:**

Early treatment is critical to improve eating disorder prognosis. Single session interventions have been proposed as a strategy to provide short term support to people on waitlists for eating disorder treatment, however, it is not always possible to access this early intervention. Conversational artificial intelligence agents or “chatbots” reflect a unique opportunity to attempt to fill this gap in service provision. The aim of this research was to co-design a novel chatbot capable of delivering a single session intervention for adults on the waitlist for eating disorder treatment across the diagnostic spectrum and ascertain its preliminary acceptability and feasibility.

**Methods:**

A Double Diamond co-design approach was employed which included four phases: discover, define, develop, and deliver. There were 17 participants in total in Australia; ten adults with a lived experience of an eating disorder and seven registered psychologists working in the field of eating disorders, who participated in online interviews and workshops. Thematic and content analyses were undertaken with interview/workshop transcriptions with findings from the previous phase informing the ideas and development of the next phase. A final prototype of a single session intervention chatbot was presented to the participants in the deliver phase.

**Results:**

Thematic and content analyses identified four main themes that were present across the four phases of interviews/workshops: conversational tone, safety and risk management, user journey and session structure, and content.

**Conclusions:**

Overall, the feedback on the single session intervention chatbot was positive throughout the Double Diamond process from both people with a lived experience of an eating disorder and psychologists. Incorporating the feedback across the four themes and four co-design phases allowed for refinement of the chatbot. Further research is required to evaluate the chatbot’s efficacy in early treatment settings.

## Abstract (plain language summary)

The earlier a person can access treatment for eating disorder symptoms, the better their chance of recovery. However, people are often waiting for long periods of time to access eating disorder care. Single session interventions have been proposed as a way of providing early treatment. These interventions are conducted as once off sessions to encourage people to make positive changes to their mental health. However, it is not always possible to access these interventions. Chatbots are digital technology that have the ability to have conversations with human users which could fill this gap. As such, the aim of this study was to co-design and develop a chatbot capable of delivering a single session intervention for adults on waitlists for eating disorder treatment. Through a series of comprehensive online interviews/workshops with people with a lived experience of an eating disorder and psychologists working in the field, we found strong support for the chatbot development, but with some concerns around the chatbot’s ability to empathise with users and ensure their safety. Solutions to these concerns were co-designed and implemented. Overall, the feedback on the final prototype version of the chatbot was positive from both people with a lived experience and psychologists.

## Background

Eating disorders are complicated illnesses with high rates of morbidity and mortality coupled with low rates of early detection and intervention [1–3]. Early intervention is considered best practice as it can substantially improve eating disorder prognosis [4, 5]. Research has indicated that the more time that is spent on waitlists, the higher the chance of the patient discontinuing treatment when they do get the opportunity [6, 7]. Thus, it is highly beneficial to offer people support while they wait to receive further treatment.

Single session interventions (SSIs) have been proposed as a solution for this gap [8]. SSIs are conducted as once off sessions structured to encourage the person to make an intentional, positive change to their mental health [9]. SSIs have great flexibility—they can be designed for different therapy modalities, delivered by a range of means (trained professionals or self-help programs), and can cater to different audiences (individuals, groups, families, parents) [8]. As a result, SSIs have the potential to reach wider audiences and at a lower cost compared to multiple session treatments [8]. Schleider et al. [10] have proposed a framework for the development of mental health focused SSIs which is comprised of four elements. First, the inclusion of scientific evidence assists in normalising the person's experiences. Second, the incorporation of narratives from other people who have overcome similar challenges. Third, empowering participants to feel they are the expert in their experience. Fourth, designing “saying-is-believing” activities where the participant identifies a challenge and then reflects on what advice they would give to a friend going through the same situation [10].

In the field of eating disorders specifically, preliminary research employing a psychologist-delivered SSI for adults on waitlists for outpatient treatment has demonstrated promising results [11]. The SSI was based on elements from enhanced cognitive behavioural therapy (CBT-E), which is considered to be the first-line treatment for adults with eating disorders [12, 13]. The SSI was designed to deliver CBT-E elements such as psychoeducation, including information on “starvation syndrome”, which are the physiological and psychological effects of prolonged dietary restriction [14]. The SSI also included collaboratively designing a formulation which is a visual diagram that identifies the maintaining factors of the person’s eating disorder [15]. The study (*N* = 448) found a significant reduction in eating disorder symptoms, psychosocial impairment, and depression symptoms from the time of completing the SSI to the first scheduled treatment session [11]. However, as this study was quasi-experimental in nature, further rigorous research is required to demonstrate effectiveness. Nevertheless, to our knowledge, this is the only study that has specifically investigated the use of an SSI for eating disorders in a clinical setting.

SSIs have also been shown to be effective with non-clinical populations/non-clinical settings at reducing body image concerns and eating psychopathology [16–20]. These SSIs have employed a variety of strategies including psychoeducation [16, 18–20], imagery rescripting [17, 18], and cognitive dissonance interventions [16, 17]. In all studies involving a control group, participants who engaged with the SSI showed a comparative reduction in eating pathology [16–18, 20]. Thus, SSIs may be effective in a range of settings. However, there is a strong need to make these SSIs accessible, scalable and engaging [8].

Research on digital mental health interventions has been increasing in recent years, particularly investigations focused on conversational artificial intelligence agents or “chatbots”. Chatbots are digital technology that can be made accessible and scalable as well as conduct engaging conversations with human users [21]. Chatbots have the potential to assist with diagnosis and triage, management and screening of symptoms, and deliver content such as psychotherapy interventions in the mental health sector [22]. Research has generally indicated that mental health chatbots are positively received by users [21, 23]. To our knowledge, to date, four specific eating disorder and body image focused chatbots have been reported in the literature: *KIT* [24] (subsequently named *JEM™* )*, Alex* [25], *Tessa* [26], and *Topity* [27].

*KIT*, a rule-based chatbot (i.e., only generates responses based on predefined question-answering rules [28]), was designed to provide psychoeducational information and brief evidence-based coping strategies for individuals experiencing body image concerns and/or eating disorders as well as for people who were seeking support for someone else [24]. *Alex,* another rule-based chatbot [25], was created for when users complete an online eating disorder screener, and target the individual's motivation to engage with treatment. *Tessa* was designed to deliver StudentBodies©, an eight-session evidence-based eating disorder prevention program [29]. However, *Tessa* has undergone further development where the chatbot went from a traditional rule-based chatbot to having a generative artificial intelligence feature [30]. *Topity* [27], a rule-based chatbot which also used gamification, was created to deliver state-based micro-interventions to elicit in the moment and short-term improvements for body esteem, affect, and body image self-efficacy.

The above chatbots all offer a range of evidence-based supports for people who have body image concerns and eating disorder pathology and are predominantly focused on pre-help seeking or connecting the user with help services. To our knowledge, none of these chatbots were designed with an SSI focus. There is an opportunity to specifically bridge the gap between when a person is first referred for eating disorder treatment to when they see a health professional for the first time. With a considerable rise in people seeking treatment for eating disorders and subsequent increases in waitlist times [31], there is a critical need for a resource that is evidence-based to provide more timely support for those seeking treatment.

Nevertheless, with technology rapidly developing, it is crucial that advances in the field maintain ethical and safe conduct [32]. For example, the aforementioned new generative artificial intelligence feature of *Tessa* chatbot led to the provision of harmful dieting and weight loss advice [30]. Thus, careful consideration is required in the development of a digital resource as there is potential of significant harm if the technology malfunctions [33]. It is recommended that a multidisciplinary team approach is implemented involving experts from different disciplines, such as mental health clinicians, researchers, developers, individuals with lived experience and ethicists, to ensure that appropriate safeguards are in place [32].

In sum, we aimed to co-design and develop a novel chatbot to deliver an SSI to adults on the waitlist for eating disorder treatment across the eating disorder diagnostic spectrum with people with a lived experience of an eating disorder, psychologists working in the field of eating disorders as well as researchers, developers and ethicists (the authors). Given the previous implementation of a psychologist delivered SSI in an eating disorder clinical setting based on CBT-E principles, we chose the broad overarching SSI framework proposed by Fursland et al. [11] as the basis for adaptation for our chatbot SSI. As the final component of this co-design and development study, we aimed to assess preliminary acceptability and feasibility of a prototype version of the chatbot.

## Method

### Participants

The study was open to participants who were aged 16 and over and living in Australia. People with a personal lived experience of an eating disorder, and in recovery for at least 2 years, were recruited by email newsletters from eating disorder support organisations in Australia. The lived eating disorder experience and recovery status was allowed to be defined by the participants themselves. Online advertisements were placed on websites and social media accounts for health professional bodies in Australia to recruit participants who were registered psychologists working in the eating disorder field. Potential participants completed an online expression of interest form linked to the email newsletters/advertisements and were then invited by author G.S. via email to participate in the online interviews/workshops based on availability. At the commencement of each interview/workshop, verbal consent from each participant was confirmed (after previous written consent was provided). Some of the psychologist participants were known to author G.S. prior to the study given a smaller size of the eating disorder psychologist field in Australia. In these instances, another author (B.D., K.d.B.) obtained the consent prior to study participation to avoid any potential perceived coercion. The study recruited a total of 17 participants. Ten individuals (eight women and two gender diverse people, aged 21 to 59 years) had a past lived experience of an eating disorder (anorexia nervosa:* n* = 3, bulimia nervosa: *n* = 4, binge eating disorder: *n* = 2, other specified feeding or eating disorder: *n* = 3, where participants could nominate more than one diagnosis) and seven registered psychologists ranging from early to late career (all women, aged 25 to 63 years) working in the eating disorder field in private outpatient psychology clinics. All participants were reimbursed with a $30 AUD online gift voucher for interview/workshop participation. This project was approved by the Monash University Human Research Ethics Committee (MUHREC ID 31812).

### Data collection

**Fig. 1:**
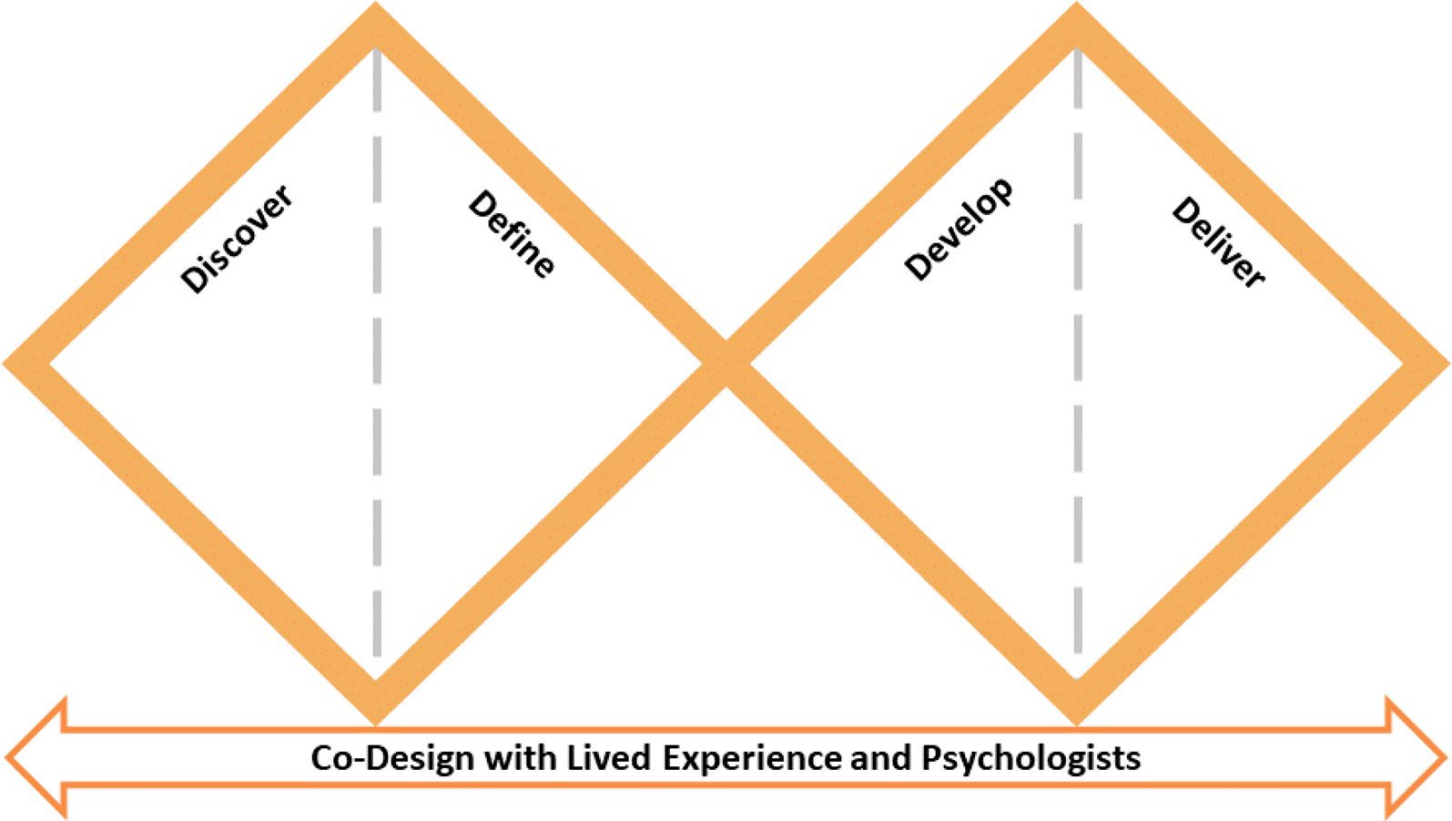
Double Diamond strategy utilised in the study adapted from [34]

The co-design data was collected in four phases according to the Double Diamond approach [34] (see Fig. 1). Broadly, the four phases aim to (1) understand the challenges faced by the target population (discover), (2) explore novel ways to address the challenges (define), (3) decide on the optimal way to implement these solutions (develop) and test and refine the optimal solution (deliver). The initial phase (discover) of one-to-one interviews was conducted by authors G.S. (PhD in clinical psychology) and R.M. (PhD in computing science) and in phases two (define), three (develop), and four (deliver), data was collected from co-design workshops led by author G.S. and supported by authors B.D. (Masters in clinical psychology), J.X. (PhD in information systems), P.S. (Masters in computer science and technology), A.N.F. (BSc[Hons] in science) and K.d.B. (PhD in clinical psychology). Note all researchers who conducted the interviews and workshops identified as women. For the workshops, lived experience participants and psychologists were grouped separately. Such an approach was adopted to allow lived experience participants to speak freely about any negative treatment experiences with health professionals they had encountered, including with psychologists.

A semi-structured question guide for each of the four phases of co-design was developed by the authors which explored participants' perspectives, requested feedback and recommendations on ideas, and offered the opportunity for participants to share their expertise. This allowed for exploration in discussion and for follow-up questions to be asked for more in-depth descriptive data. Seven one-to-one interviews were conducted for the discover phase, and four workshops were run for each of phase two (define), three (develop) and four (deliver) of the Double Diamond process where data saturation was reached for each phase. New participants were introduced at each of the four co-design phases to ensure a diverse range of perspectives and feedback. The initial interviews and subsequent workshops were conducted and audio recorded using the digital platform Zoom, whereby the discover phase interviews (April 2022) lasted from 35 to 50 min (*M* = 45 min), the define phase workshops (August 2022) from 45 to 66 min (*M* = 53 min), develop workshops (November 2022) from 63 to 77 min (*M* = 68 min) and the deliver phase workshops (May 2023) from 52 to 77 min (*M* = 68 min).

### Design process

**Fig. 2:**
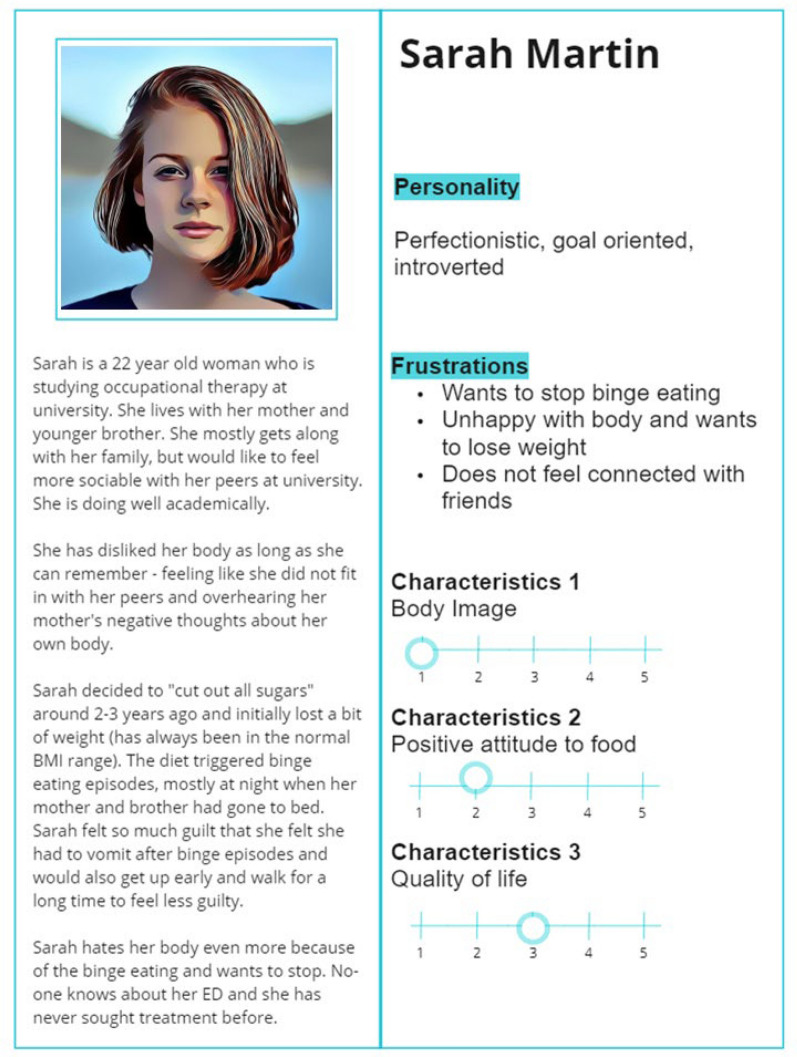
An example of a co-designed persona, “Sarah Martin”, employed in the Double Diamond process

According to the Double Diamond approach, the objective of the discover phase was to understand the level of acceptability by the different participant populations for the concept of a chatbot delivering an SSI to adult patients waiting for eating disorder treatment. Additionally, the perspectives of the participants were explored particularly focusing on the advantages and disadvantages of this proposed idea. In the define phase, key ideas and topics that emerged from the discover phase were refined. Topics explored in the workshops included proposed characteristics of a typical user of the chatbot (in order to generate personas of multiple genders, see Fig. 2 as an example), the content of the chatbot, and gaining perspectives on preliminary examples of the chatbot dialogue.

**Fig. 3:**
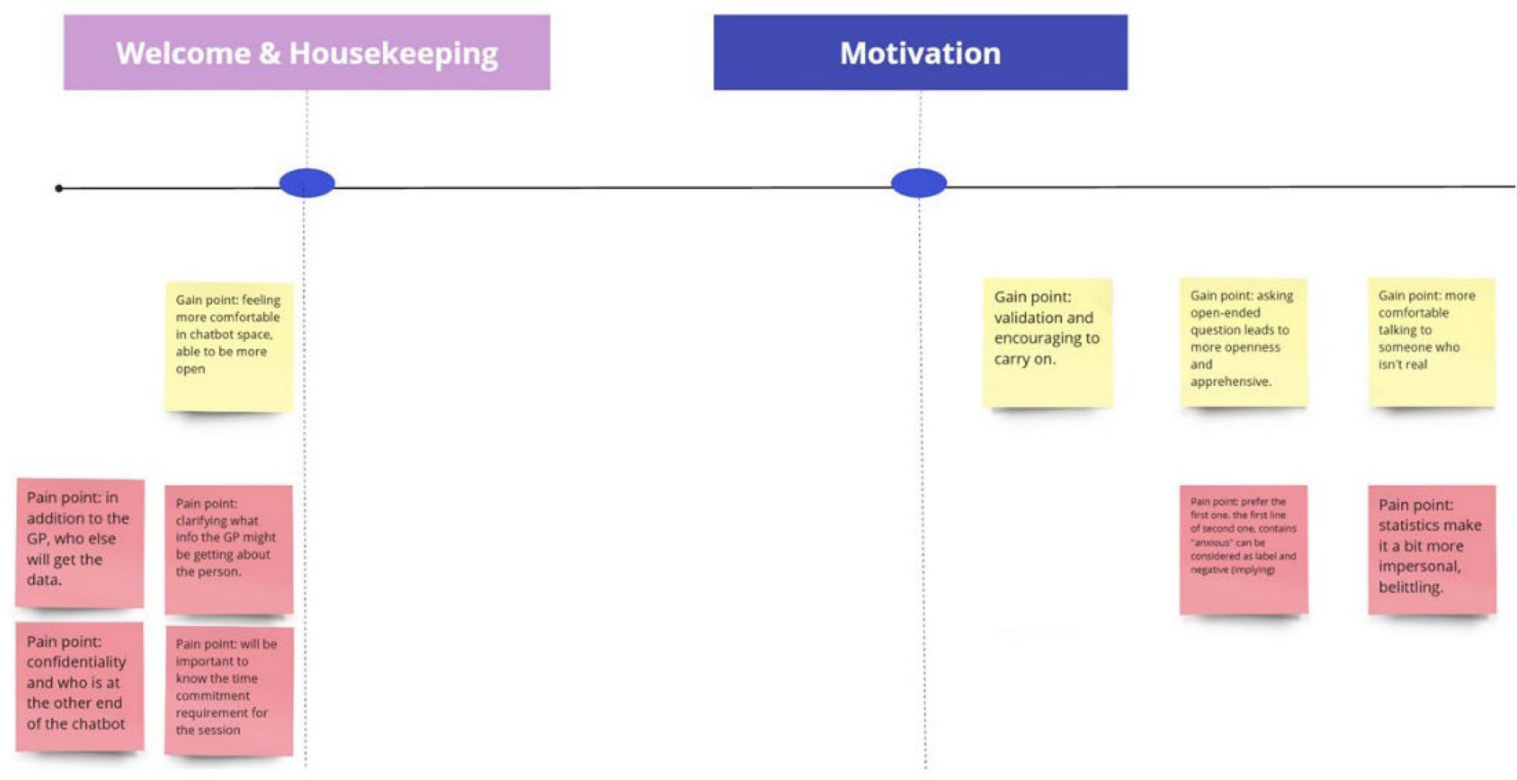
A screenshot of an example portion of the SSI user journey shown in the develop workshops with participant nominated gain and pain points displayed in yellow and red post-its respectively. The seven-sections of the SSI user journey were: (1) Welcome and Housekeeping, (2) Building Motivation, (3) Symptom Clarification, (4) Psychoeducation, (5) Formulation, (6) Regular Eating, and (7) Closing

In the develop phase workshops, the co-designed personas were employed to guide participants through the proposed user SSI journey and seek feedback in the form of gain and pain points (Fig. 3). Brief mock-ups of chatbot dialogue along the user journey were also presented for feedback.

**Fig. 4:**
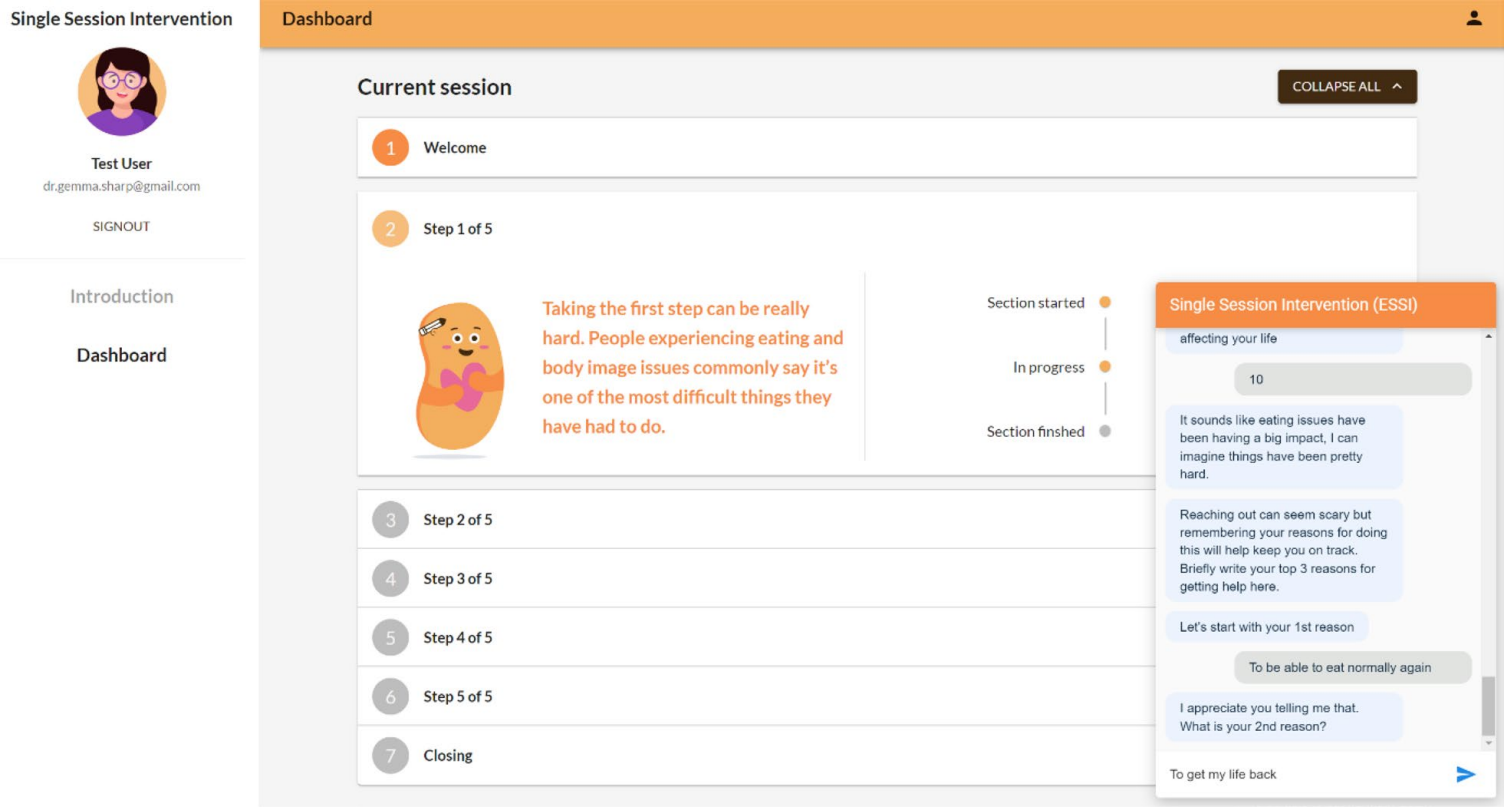
Screenshot from the final prototype of the SSI chatbot web interface presented to the participants in the deliver phase. The specific chat interface is shown in the bottom right of the screen. The web-based user dashboard (displayed in the background) shows individual user progress through the seven SSI sections and displays short summaries of the chat for each section. The co-designed SSI chatbot character is shown on the dashboard under “Step 1 of 5” (with the 5 steps being: Building Motivation, Symptom Clarification, Psychoeducation, Formulation, Regular Eating) and is also included in the chat interface

The aim of the deliver phase was to implement the finalised chatbot design and preliminarily test the developed working prototype. Powered by the Google Dialogflow platform [35], this rule-based chatbot followed a pre-determined set of conversational rules and could only provide pre-programmed responses designed by the authors [28]. There were no machine learning techniques employed in the chatbot’s design. The chatbot captured user inputs and retained individual conversation history, accessible exclusively by the respective user. Such an approach was used to prioritise safety and security. Subsequently, the chatbot was integrated into a specifically designed hosting web interface to enhance user interactions throughout the linear (forward direction only) seven-section user journey which took about 30 min to complete in total. The start of the chatbot session focused on validating the decision of the user to seek treatment and enhance motivation for treatment. By the end of the session, the user had been introduced to the concept of regular eating for recovery [15] and to set a goal to work on while waiting for in-person treatment on this topic (e.g., eat breakfast every day or consume a type of food they had previously been avoiding). Usability testing was conducted in the workshops and participants provided final feedback on the prototype (Fig. 4).

### Data analysis

The qualitative data collected from the co-design interviews and workshops across the four phases was analysed using a codebook thematic analysis approach [36, 37] along with elements of content analysis [38], which, together, allow common patterns and themes within a dataset to be identified [39, 40]. A confidential automated transcription service was used to transcribe the audio recordings from all interviews/workshops. The transcriptions were read and checked for errors and initial ideas were noted by G.S., B.D. and K.d.B.. The transcripts for the lived experience participants and psychologist participants were analysed together and were given equal weighting. Transcripts were initially analysed, and codes were generated using a deductive thematic analysis approach, guided by the questions/ideas of each interview/workshop. Transcripts were then analysed and codes were generated using an inductive thematic analysis approach, identifying and subsequently grouping codes within each question/idea [40]. Phases 2 to 4 drew from elements of inductive content analysis to categorise participant responses to specific ideas or components of the single session/chatbot design [38]. Codes were grouped into overarching themes by G.S., K.d.B., and B.D.. These themes were used to inform development of the chatbot for the next phase of the study. Upon the completion of all four phases, the codes and themes were reviewed by G.S. and B.D., who refined and named the themes which were agreed upon by all authors. Author G.S. oversaw all data analysis.

### Reflexivity statement

I, Gemma Sharp, conducted the main analyses and acknowledge the subjectivity and context that may have influenced the research process. While drawing on my previous research and clinical psychologist experience in early intervention and chatbot design in the context of eating disorders to design this study, I remained mindful to not constrain the research scope or limit participants from the provision of diverse  perspectives. To control the potential impact of my prior research and clinical experience on the interpretation of interview/workshop data, we involved research assistants/post-doctoral researchers skilled in qualitative research with a less extensive background in early intervention and chatbot development in eating disorders specifically in the design of the interviews/workshops (authors B.D., K.d.B., R.M., J.X.) as well as in the process of secondary coding (authors B.D., K.d.B.). This approach introduced new perspectives and I was considerate of all undue influences from my previous research.

## Results

Thematic analysis showed four major themes present through almost all phases of the co-design process: (1) conversational tone, (2) safety and risk management, (3) user journey and session structure, and (4) content (see Table 1).

**Table 1:** Themes and quotes from the four-phase co-design process

Phase	Participant group	Quote number	Example quote
Theme: Conversational tone			
Discover	Psychologist	1	“Is validation from a chatbot and hearing your experience the same as being in the company of another human being? Does it have empathy, and, I don't know the answer to this, but does it have empathy in the same way a human would?” [Participant 4, Woman, Aged 55]
	Psychologist	2	“I wonder what their level of engagement would be like, with a chatbot because they're not getting that human interaction. And I think that human interaction that provides all of that real validation and understanding, I think that might be the difference.” [Participant 3, Woman, Aged 34]
Define	Psychologist	3	”So, trying to validate them in terms of making them feel not alone. And I wonder if that’s one of the ways to go.” [Participant 5, Woman, Aged 25]
	Lived experience	4	“Openness is really quite powerful, I think. And that leads into kind of motivating them more and wanting them to kind of seek more help. So, keeping their rhythm going for them.” [Participant 11, Woman, Aged 35]
Develop	Psychologist	5	“I wonder if we can say, ‘This might be really challenging, but the good news …’ you can sprinkle a bit of hope in there.” [Participant 5, Woman, Aged 25]
	Lived experience	6	“I like how it makes him [persona] see the bigger picture of things. And once again, yeah, just that he's not alone.” [Participant 3, Gender Diverse Person, Aged 21]
Deliver	Psychologist	7	“I guess, like sort of real-time feedback from what the consumers have said, to sort of spit it back out, because I think that's important and it shows users that what they are saying is important, and sort of just validates what they're going through.” [Participant 5, Woman, Aged 25]
	Psychologist	8	“It's offering some validation, some empathy there straightaway without it feeling inauthentic.” [Participant 4, Woman, Aged 55]
	Lived Experience	9	“I like the goal part that it makes you… encourages a small change, or something that you can do. You don't need to worry.” [Participant 11, Woman, Aged 35]
Theme: Safety and risk management			
Discover	Psychologist	10	“How does it cope with crises? So something like ‘I really want to die’, or ‘I've got a medical emergency’. So, psychiatric or medical emergencies? I don't know how it would deal with that and where the risk would lie in that.” [Participant 4, Woman, Aged 55]
	Lived experience	11	“If you say to them, ‘I'm struggling or this isn't right. What do I do?’ the chatbot’s not going be able to support you … Or, like wouldn't know the severity of what's happening or wouldn't be able to give you the right advice.” [Participant 9, Woman, Aged 27]
Define	Psychologist	12	“I think a chatbot can assess risk, but what does the chatbot do if there is risk? What are the consequences of assessing risk?” [Participant 4, Woman, Aged 55]
	Lived experience	13	“I was actually curious, say somebody starts to really explore the app and identifies certain symptoms that are occurring at the moment and we are looking at a quite serious situation. Is there the ability to give that person the choice to connect in with somebody, for example, that they’ve clicked into, or can it recommend going to emergency or contacting your GP [general practitioner]?” [Participant 12, Woman, Aged 46]
Develop	Psychologist	14	“Just making sure that the person using the services is aware that this isn't a crisis support, sort of service. And if they are feeling really distressed immediately that they should call triple zero [emergency phone number] or go to the local emergency department.” [Participant 5, Woman, Aged 25]
Deliver	Psychologist	15	“I like that it's got the get urgent help all the way through.” [Participant 2, Woman, Aged 58]
	Psychologist	16	“It terminates the session, if you're symptomatic… that it seems appropriate. There's a sense of urgency around it, there's no point in being in this chat if you are symptomatic. I like that it raises some sort of alert with the person in the session. You should seek medical advice if you experience these things.” [Participant 4, Woman, Aged 55]
	Lived experience	17	“Oh, that's awesome, it comes up with heaps of crisis service references.” [Participant 9, Woman, Aged 27]
Theme: User journey and session structure			
Define	Psychologist	18	“I think as long as you're clear about what's being sent to other people, the GP and the psychologist.” [Participant 4, Woman, Aged 55]
	Lived experience	19	“I think it’s really important as well to state at the start that it’s a robot.” [Participant 9, Woman, Aged 27]
	Lived experience	20	“You can always come back and check in. If you’ve had enough, you know, that sort of just coming back every now and then saying, you can come in and check in, do you need a break?” [Participant 12, Woman, Aged 46]
Develop	Psychologist	21	“Looks like the sort of flow you would have in an in-person session. So, it very much follows that.” [Participant 4, Woman, Aged 55]
	Psychologist	22	“I think that first initial invitation is very important.” [Participant 1, Woman, Aged 63]
	Lived experience	23	“I think it flows really, really well.” [Participant 11, Woman, Aged 35]
Deliver	Psychologist	24	“I didn't find it difficult at all. And I'm, I'm a moron when it comes to this sort of stuff.” [Participant 2, Woman, Aged 58]
	Psychologist	25	“The more I'm going through this, I'm thinking it is nice to offer the opportunity to have a break rather than have people just disengage. I think that's quite nice to say, ‘Have you had enough of this right now have a break and come back’”. [Participant 4, Woman, Aged 55]
	Lived experience	26	“I like it. I really like how it kind of checks in. So, I'm at the point where it's going ‘Are you okay to continue?’, which is really good.” [Participant 11, Woman, Aged 35]
	Lived experience	27	“If I was completing this as a client, I wouldn't feel like I was just talking to a literal chat, I would feel like, what I'm saying is going towards something purposeful and meaningful.” [Participant 9, Woman, Aged 27]
	Lived experience	28	“The way that it ends, it feels unfinished.” [Participant 16, Woman, Aged 29]
	Lived experience	29	“I think I'm at the end, I've got to the happy chatbot character….does that mean I have gone to the last section?” [Participant 11, Woman, Aged 35]
	Psychologist	30	“Just a bit of extra validation at the end, just to sort of tie everything up.” [Participant 5, Woman, Aged 25]
	Psychologist	31	“One thing I think would be nice at the end, too, is reminding the person of their reasons for recovery.” [Participant 4, Woman, Aged 55]
	Lived experience	32	“I really like how, if you're feeling motivated giving people that option of further education.” [Participant 16, Woman, Aged 29]
Theme: Content			
Discover	Psychologist	33	“So, if there is a capacity, ultimately down the track for something that's a bit interactive would be helpful.” [Participant 4, Woman, Aged 55]
Define	Psychologist	34	“I think that you can’t be too rushed and I think it has to be in sort of bite-sized chunks. That’s easily understandable. I would imagine you would lose that sort of jargon.” [Participant 5, Woman, Aged 25]
	Psychologist	35	“I think having some sort of animation or video would really grab the attention of consumers.” [Participant 5, Woman, Aged 25]
	Psychologist	36	“I can appreciate the difficulty of creating a diagram like that, that's personalised and collaborative.” [Participant 4, Woman, Aged 55]
	Psychologist	37	“So, you don’t want the chatbot to suddenly come up with a diagram that it draws out and doesn’t wait for you to keep up or doesn’t offer you an opportunity for input. So, it’s very important it would be a collaborative part of the session.” [Participant 4, Woman, Aged 55]
	Lived experience	38	“I think it can be so overwhelming to get too much information at once. So, it’s navigated at a time that suits them best. So, I think keeping it as simplified as possible.” [Participant 12, Woman, Aged 46]
Develop	Psychologist	39	“You want to keep it fairly simple, I think. I'm trying to put myself in the position of a client seeing this for the first time, I'd be like, ‘Whoa’”. [Participant 4, Woman, Aged 55]
	Psychologist	40	“I did like the formulation section. So, I'm not saying delete it. I'm just saying if it's too hard, there is an alternative.” [Participant 1, Woman, Aged 63]
	Psychologist	41	“The chatbot could have a template, for example, with the boxes already and then the chatbot kind of does a guide of like, ‘how do you talk about your body image concerns?’” [Participant 6, Woman, Aged 52]
Deliver	Psychologist	42	“It's a really nice succinct little way of talking about it and the messages were really clear. You know, the key messages were highlighted but were also clear, which was great.” [Participant 2, Woman, Aged 58]
	Lived experience	43	“I love the content. I think it's fantastic. And it's very succinct and easy to read as well, it's relevant. If I was completing this as a client, I wouldn't feel like I was just talking to a literal chat, I would feel like what I'm saying is going towards something and purposeful, meaningful.” [Participant 9, Woman, Aged 27]
	Lived experience	44	“I liked it. It's interactive. And you have a record of what you said. The videos make a good break from answering questions, you just get a bit of information, but it's not like a lecture. And then the information is summarised, which again, is really helpful.” [Participant 16, Woman, Aged 29]
	Lived experience	45	“I think that could be actually very helpful. And I think the younger generation now that I'm teaching kind of young females as well, with my work, I find that this is kind of their preferred method to tell you the truth anyway, they're so tech savvy, and used to communicating kind of with computers before people.” [Participant 8, Woman, Aged 42]

### Theme: Conversational tone

The tone of the chatbot was discussed extensively in all phases of the co-design process in both the lived experience and psychologist groups. Participants reported the importance for the chatbot to be able to provide therapeutic strategies, such as validation, promote a feeling of hopefulness, and normalisation. In the discover phase, the participants in both groups reported concerns that interactions with the chatbot would not provide the same experiences as interactions with a human health professional, and whether this would be a barrier to user engagement (Quotes 1 and 2). In the define and develop phases, the research team worked closely with the participants to develop the tone of the chatbot by working with mock-ups of the chatbot’s dialogue. Participants discussed how therapeutic strategies could be adapted and delivered by a chatbot. Suggestions from the psychologist workshops included normalising the feeling of isolation and revising the dialogue to include elements of hope (Quotes 5 and 6). In contrast, the lived experienced groups reported the significance of creating an environment where the user felt they could be open and honest with the chatbot (Quote 4). Participant feedback in the deliver phase showed that the prototype chatbot was able to provide therapeutic strategies throughout the session. Specifically, one psychologist participant reported that the chatbot was able to provide empathy and validation in an authentic way in response to the user reporting that their body image concerns and eating behaviours were negatively impacting their life (Quote 8). This was further supported by two other participants who reported that the prototype chatbot was able to reflect back to users what they had said (Quote 7) and encourage positive change (Quote 9).

### Theme: Safety and risk management

The psychologists and the lived experience participants identified in the discover phase that the chatbot would require the ability to manage medical and psychiatric risk. Participants from both groups reported uncertainty surrounding whether the chatbot would be able to manage crisis situations (Quote 10) and provide appropriate support (Quote 11) for medical and psychiatric risk. These concerns were stated again in the define phase, where psychologists reported that the chatbot may be able to assess risk, however, they were concerned about the implications of a chatbot assessing risk and the subsequent actions a chatbot could take (Quote 12). The lived experience participants reported the importance of being transparent with the user at the start of the session, where if risk was identified additional services (e.g., treating medical doctor) would be alerted due to safety concerns (Quote 13). This was also raised in the psychologist workshops in the develop phase, where the participants reported that it needed to be explicitly outlined to the user that the chatbot was not a crisis service from the start of the conversation (Quote 14). In the deliver phase, participants were shown a prototype of the chatbot’s risk detection strategies and alert systems. Both groups of participants reported their concerns were alleviated around risk as sufficient access to crisis resources were available throughout the session as well as the termination of the session when medical or psychiatric risk was detected (Quotes 15, 16 and 17).

### Theme: User journey and session structure

In the define and develop phases, the proposed user journey of the SSI as delivered by the proposed chatbot was presented to both groups of participants. Both groups of participants reported approval of the seven-section session structure (Quotes 21 and 23). It was important to both groups of participants that the start of the session had an impact on the user. Participants reported that the start of the session had to be balanced between being friendly and inviting while also stating the parameters of the session, for example, a privacy agreement to communicate to the user who would have access to the session information, and stating to the user that the chatbot was not a real person (Quotes 18 and 19). Additionally, during the define and develop phases, the lived experience participants reported the importance for the chatbot to check in on the user’s psychological well-being throughout the session and to offer a break to allow for the user to navigate the session at their own pace (Quotes 20). In the deliver phase, participants reported that the prototype chatbot session was easy to navigate and complete (Quote 24). Participants reported that they liked how the chatbot checked in with the user and offered breaks (Quotes 25 and 26). Specifically, a lived experience participant reported that the design of the session felt relevant and purposeful (Quote 27). Some of the participants reported that the end of the session felt “unfinished” (Quote 28). Participants reported uncertainty about whether they had finished the session or not (Quote 29). Participants reported that the inclusion of additional validation statements (Quote 30), reminders of what was covered in the session (Quote 31), as well as providing opportunity for further education (Quote 32), would assist in providing a clearer ending to the session.

### Theme: Content

The content of the SSI chatbot and how to present the information was discussed at length across all four phases. In the discover phase, participants reported that the SSI chatbot needed to have interactive elements to enhance engagement (Quote 33). In the define phase, the researchers explored how to present the content with the participants. Lived experience and psychologist groups reported that the information provided needed to be in lay-person language and small amounts of information presented at a time so that the user was not overwhelmed with information (Quotes 34 and 38). Additionally, both groups stated that including short videos in the session, including those featuring people with a lived eating disorder experience, would help maintain the user’s attention (Quote 35).

A typical component of eating disorder treatment, CBT-E in particular, is to introduce the patient to a formulation which is a diagram of how their eating disorder symptoms are connected and how this turns into a pattern of repetitive behaviour. This was identified as being the most difficult task for the chatbot to support and was discussed at length during the define, develop, and deliver phases. Participants reported that this task had to be collaborative with the user (Quote 39). The develop phase focused on how to simplify the formulation task (Quotes 39 and 40). Additionally, the psychologist participants stated that a template could be provided to the user to make the task easier to complete (Quote 41). In the deliver phase, the participants were shown how the prototype chatbot conducted the formulation task using templates and both groups of participants approved of this strategy (Quote 42).

Overall, the prototype chatbot content was widely accepted in the deliver phase by both the psychologist and lived experience participants. Specifically, a participant reported that the messages of the intervention were clear and succinct (Quote 43). Participants stated that the interactive elements of the intervention provided a break from using the chat feature (Quote 44) and the user’s contribution to the session felt purposeful (Quote 43). Participants reported that the chatbot could be very helpful, especially for young adult users who were more familiar with technology (Quote 45).

## Discussion

This study aimed to co-design, with multiple groups, a novel chatbot capable of delivering an SSI for adults on the waitlist for eating disorder treatment across the diagnostic spectrum. Analysis of the qualitative data yielded four major themes across the four phases of the study; conversational tone, safety and risk management, user journey and session structure, and content. Across these themes, participants provided overall positive feedback, thus, the chatbot was deemed preliminarily acceptable and feasible.

The prototype chatbot appeared to be successful in replicating some of the key elements of conversational tone to a health professional. This was deemed essential by people with a lived experience and psychologists in the discover phase and throughout the entire co-design process. By being able to achieve this tone in the final prototype, it may possibly assist the user in making the positive changes addressed within the SSI. Discussion about management of safety and risk focused on potential limitations of the chatbot, for example, that the chatbot was not a real person, not a crisis service, and if risk was detected, it would be beyond the chatbot’s abilities to alert appropriate services. This influenced the final prototype where: disclosure statements specifically addressing the above concerns were provided before the SSI began, appropriate crisis service contact details were provided when risk was detected, or the SSI terminated with the instruction to go to the nearest emergency department of a hospital with an alert sent to the user’s treating medical doctor. Chatbot user safety was further enhanced through the choice of a rule-based chatbot (i.e., can only provide predetermined responses) and this decision was strongly supported by both lived experience and psychologists. Some of the participants specifically mentioned concerns around documented harms of using generative artificial intelligence in a mental health setting (e.g., *Tessa* chatbot offering inappropriate dieting and weight loss advice in an eating disorder context [30]) and so were keen to make the SSI chatbot as safe as possible. People with a lived experience and psychologists approved the user journey and session structure of the final prototype. The lived experience participants found the chatbot very easy to use and navigate, whereas some extra instruction was required for psychologists. Both groups readily accepted the content of the chatbot most likely as the overarching broad framework originated from a pre-existing in-person SSI employing CBT-E elements [11]. Overall, both groups were highly accepting of the prototype chatbot.

To our knowledge, two other eating disorder focused chatbots (although not specifically focused on early treatment/intervention) reported qualitative themes in their development research which shared similarities with the current study. Our conversational tone in the current study was similar to the tone theme in the development of *Alex* [25] and the tone subtheme for *KIT* [24]. Across these themes, users reported that engagement in the chat increased the likelihood of making positive changes [25], and that it was important for the chatbot to use therapeutic strategies, such as normalisation [24]. Additionally, the user journey and session structure theme in the current study was reflected in the ease of use theme in *Alex* [25] and the flow theme in *KIT* [24], where ease of use and navigation of the chatbot’s conversation were considered highly important. Interestingly, the safety and risk management theme identified in the current study was not reported in the development of *KIT* [24] or *Alex* [25], nor was it identified in a scoping review of user experiences of mental health chatbots [28]. Potentially, as our chatbot was designed to be integrated at the start of clinical treatment, safety and risk management was deemed even more critical for user safety.

As stated above, the development of our chatbot delivered SSI was based on the broad overarching framework of a psychologist delivered in-person SSI [11] which targeted assessment of eating disorder symptoms and provided educational information on eating disorders. For this in-person offering, ~ 30% of the sample did not attend the SSI [11]. Providing an SSI through a digital platform could potentially increase the percentage of people engaging with an SSI owing to ease of access [41], which may, in turn, give more time back to health professionals to attend to higher risk clinical matters. Attending in-person treatment for an eating disorder for the first time can be a daunting process and so a first session with a chatbot could potentially help to ease some anxiety around attending further in-person treatment [24]. However, future research is required to determine the popularity of our SSI chatbot offering in real world clinical settings.

The design of the current study’s chatbot SSI also aligned closely with Schleider et al.’s [10] broader mental health SSI framework. Specifically, scientific evidence was included, such as information about the risks of eating disorders and on starvation syndrome [14]. Incorporation of people with lived experience through short video clips echoed the element of including a personal narrative. Additionally, the chatbot SSI was designed so that users took an active role in the session by bringing their personal experiences into the content of the session, particularly the formulation exercise.

## Strengths and limitations

The main strength of this study was the extensive co-design process with groups of people with lived experience and psychologists following the Double Diamond approach [34]. This human-centred design strategy brought a broader range of expertise to the design team (researchers, developers, ethicists) and allowed for the end user to be placed at the centre of the design process. Thus, the final product has greater potential to meet the needs of the user. This could be seen by the high level of acceptability with the participants with the final prototype. Additionally, the content of the SSI included elements of CBT-E, thus ensuring the chatbot is providing a high level of evidence-based support in an eating disorder context [12].

There were, however, several limitations in the current study. First, there was little diversity in demographic characteristics within the sample from a lived experience perspective (e.g., mostly young adult women). Therefore, it may be difficult to generalise the findings to the diversity of people impacted by eating disorders. Nevertheless, the personas developed to aid in the co-design were of multiple genders and ages to attempt to reflect this diversity. Another limitation was that the co-design interviews/workshops were conducted with some members of the authorship team (all women) and so participants may have avoided providing very negative feedback knowing that the people directly responsible for the chatbot design were present. Additionally, an inclusion criterion for the lived experience participants was that the participants had to be recovered from their eating disorder. There may be a bias as these participants possibly had more positive treatment experiences. Future research is certainly needed to investigate the willingness of people on waitlists to use the chatbot in real world settings and its effectiveness in promoting early improvements in eating disorder symptomatology. Finally, we could have potentially involved other key stakeholders in the co-design process (e.g., other types of health professionals involved in eating disorder care such as dietitians and carers/supporters of adults with an eating disorder) as well as collected more information on career length of the psychologist participants. Nevertheless, in most settings, this SSI interaction is generally limited to a one-to-one interaction with psychologists and their adult patients. In spite of this, there has been more suggestion recently to involve other key people, like carers/supporters, routinely in adult eating disorder treatment [42].

## Conclusions

The current study provides preliminary evidence for the acceptability and feasibility for a chatbot co-designed to deliver an SSI to adults on the waitlist for eating disorder treatment. To the best of our knowledge, this is a world-first waitlist SSI chatbot for eating disorders across the diagnostic spectrum. Overall, participants with lived experience and psychologists working in the field of eating disorders provided positive feedback on the conversational tone, safety and risk management, user journey and session structure, and content of the chatbot. If proven effective in future research, this chatbot has the potential to provide access to earlier treatment by filling crucial gaps in eating disorder care and to help people to recover more quickly from serious illness.

## Data Availability

The datasets generated and analysed during the current study are not publicly available under the participant confidentiality conditions of ethics approval from the Monash University Human Research Ethics Committee.

## Abbreviations

CBT-E: Enhanced cognitive behavioural therapy
SSI: Single session intervention
